# 3D Printed frames to enable reuse and improve the fit of N95 and KN95 respirators

**DOI:** 10.1186/s42490-021-00055-7

**Published:** 2021-06-07

**Authors:** Malia McAvoy, Ai-Tram N. Bui, Christopher Hansen, Deborah Plana, Jordan T. Said, Zizi Yu, Helen Yang, Jacob Freake, Christopher Van, David Krikorian, Avilash Cramer, Leanne Smith, Liwei Jiang, Karen J. Lee, Sara J. Li, Brandon Beller, Kimberley Huggins, Michael P. Short, Sherry H. Yu, Arash Mostaghimi, Peter K. Sorger, Nicole R. LeBoeuf

**Affiliations:** 1 Greater Boston Pandemic Fabrication Team (PanFab) c/o Harvard-MIT Center for Regulatory Science, Harvard Medical School, Boston, MA USA; 2 Department of Neurosurgery, University of Washington, Seattle, WA USA; 3 Harvard Medical School, Boston, MA USA; 4 Harvard Graduate School of Design, Cambridge, MA USA; 5 Harvard-MIT Division of Health Sciences and Technology, Cambridge, MA USA; 6 Department of Systems Biology, Harvard Ludwig Cancer Research Center, Harvard Medical School, Boston, MA USA; 7 Harvard-MIT Center for Regulatory Science, Harvard Medical School, Boston, MA USA; 8 Fikst Product Development, Woburn, MA USA; 9 Borobot, Middleborough, MA USA; 10 Dana-Farber Cancer Institute, MA Boston, USA; 11 Department of Radiology, Brigham and Women’s Hospital, Boston, MA USA; 12 Department of Dermatology, Brigham and Women’s Hospital, Boston, MA USA; 13 Engineering Science at Norwalk Community College, Norwalk, CT USA; 14 Department of Nuclear Science and Engineering, Massachusetts Institute of Technology, Cambridge, MA USA; 15 Department of Dermatology, Yale University School of Medicine, New Haven, CT USA; 16 Harvard Program in Therapeutic Science, Harvard Medical School, Boston, MA USA

**Keywords:** COVID-19, pandemic response, personal protective equipment (PPE), N95 respirators, KN95 masks, 3D printing, filtering face piece (FFP) respirator, mask frames, prototyping, occupational health

## Abstract

**Background:**

In response to supply shortages caused by the COVID-19 pandemic, N95 filtering facepiece respirators (FFRs or “masks”), which are typically single-use devices in healthcare settings, are routinely being used for prolonged periods and in some cases decontaminated under “reuse” and “extended use” policies. However, the reusability of N95 masks is limited by degradation of fit. Possible substitutes, such as KN95 masks meeting Chinese standards, frequently fail fit testing even when new. The purpose of this study was to develop an inexpensive frame for damaged and poorly fitting masks using readily available materials and 3D printing.

**Results:**

An iterative design process yielded a mask frame consisting of two 3D printed side pieces, malleable wire links that users press against their face, and cut lengths of elastic material that go around the head to hold the frame and mask in place. Volunteers (n = 45; average BMI = 25.4), underwent qualitative fit testing with and without mask frames wearing one or more of four different brands of FFRs conforming to US N95 or Chinese KN95 standards. Masks passed qualitative fit testing in the absence of a frame at rates varying from 48 to 94 % (depending on mask model). For individuals who underwent testing using respirators with broken or defective straps, 80–100 % (average 85 %) passed fit testing with mask frames. Among individuals who failed fit testing with a KN95, ~ 50 % passed testing by using a frame.

**Conclusions:**

Our study suggests that mask frames can prolong the lifespan of N95 and KN95 masks by serving as a substitute for broken or defective bands without adversely affecting fit. Use of frames made it possible for ~ 73 % of the test population to achieve a good fit based on qualitative and quantitative testing criteria, approaching the 85–90 % success rate observed for intact N95 masks. Frames therefore represent a simple and inexpensive way of expanding access to PPE and extending their useful life. For clinicians and institutions interested in mask frames, designs and specifications are provided without restriction for use or modification. To ensure adequate performance in clinical settings, fit testing with user-specific masks and PanFab frames is required.

**Supplementary Information:**

The online version contains supplementary material available at 10.1186/s42490-021-00055-7.

## Background

Frontline health care workers are vulnerable to infection by the severe acute respiratory syndrome coronavirus 2 (SARS-CoV-2), which causes coronavirus disease 2019 (COVID-19) [1–4]. Respiratory protection is an essential component of preventing hospital-based infections, but an unprecedented demand for N95 filtering facepiece respirators (FFRs; N95 masks) has led to severe shortages. Many institutions have been forced to look for ways to reuse masks, rely on unfamiliar makes and models, and even develop alternative forms of respiratory protection [5–8].

The US Centers for Disease Control and Prevention (CDC) recommends that healthcare workers dispose of N95 masks after each patient encounter. However, during the 2009 influenza A (H1N1) pandemic, which involved many fewer cases and deaths than the current COVID-19 pandemic, N95 respirator supplies were depleted [9–11]. In response, guidelines were developed for N95 “extended use” and “reuse” as a means to conserve supplies during shortages. Extended use is the practice of wearing the same respirator for contact with several different patients infected with the same respiratory pathogen, without disposing of the respirator between patients. Reuse refers to using the same N95 respirator after removing it (“doffing”), for instance after a healthcare worker’s shift has ended, and then putting it back on (“donning”) prior to the next patient encounter. In the current COVID-19 pandemic, there is no specific regulation limiting the number donning/doffing cycles for N95 masks [7], although previous work has found that masks consistently fail fit testing after five consecutive donnings [12]. Individual healthcare settings have therefore enacted their own policies to restrict respirator reuse and extended use [13].

In the US, surgical N95 FFRs used in healthcare are regulated by the National Institute for Occupational Safety and Health (NIOSH; part of the CDC) and the Food and Drug Administration (FDA) and must conform to standards set out in US 42 CFR part 84. Other countries have analogous regulations, including the GB2626-2006 standard for KN95 masks in China and the EN 149:2001 standard for FFP2 masks in Europe. All such masks must exhibit three essential properties: (i) efficient filtration of small particles (ii) unencumbered inhalation and exhalation when a mask is in place and (iii) snug fit to the face of a user so that all inhaled air passes through the filtering fabric. Mask reuse is often limited by the difficulty of achieving good fit as a result of breakage or degradation of elastic bands that hold the mask in place; unused masks that have been in storage for an extended period of time (e.g. in emergency stockpiles) also suffer from a loss of band elasticity and integrity. When respirators are reused, fit is also negatively impacted by degradation of nose clips and other components required to seal a mask tightly to a user’s face [14].

The aim of this study was to develop a freely available public domain design for a simple device (a mask frame) to improve the fit of N95 respirators damaged by extended storage or reuse, thereby prolonging their lifespan and increasing overall mask availability. A secondary goal was to improve mask fit for individuals who failed baseline testing, thereby increasing the number of individuals who could potentially benefit from low-cost respiratory protection. In the latter case, we focused on Chinese-made KN95 masks, which are similar in performance to N95 FFRs [15]. KN95s are increasingly available but often fail fit testing [16]. The mask frame that we developed uses readily available materials and simple 3D printing technologies (on consumer-grade machines), and can be customized to individual faces simply by bending malleable components. To simplify deployment, we sought to cover the great majority of users with as few mask frame parts as possible and settled on a solution with two frame sizes.

## Methods

### Frame design process

Development of a modular mask frame model was inspired by the work of Dr. Christopher Wiles at the University of Connecticut, who has used 3D printed frames to enable use of alternative filter fabrics such as Halyard H600 sterilization wrap [17]. We attempted to use the same relatively rigid 3D printed design to hold in place a standard 3M Model 8210 (St. Paul, MN) N95 industrial respirator. However, we found that the frame did not fit many individuals, particularly females with narrow faces. Previous research has shown that there are key facial dimensions affecting respirator fit [18]. We therefore sought to develop a frame with flexible components that could be molded by a user to assist in optimizing fit along these dimensions. The final design was the result of an iterative process, which consisted of multiple rounds of mask and prototype fit testing on volunteers (students and healthcare professionals) with design modifications made based on user feedback. Direct interaction between users and designers facilitated the process. Key features added in the iterative design process included the production of two frame sizes to improve fit to faces of different shapes, the addition of clips to help secure the mask frame to the underlying respirator and decrease the likelihood of the frame falling off during use, and modifications to the location and length of the frame head bands to make donning and doffing easier. We freely provide all designs in standard electronic formats for use by others or for further modification through the NIH 3D Print Exchange (https://3dprint.nih.gov/discover/3dpx-014725).

### Mask frame software and design

**Fig. 1:**
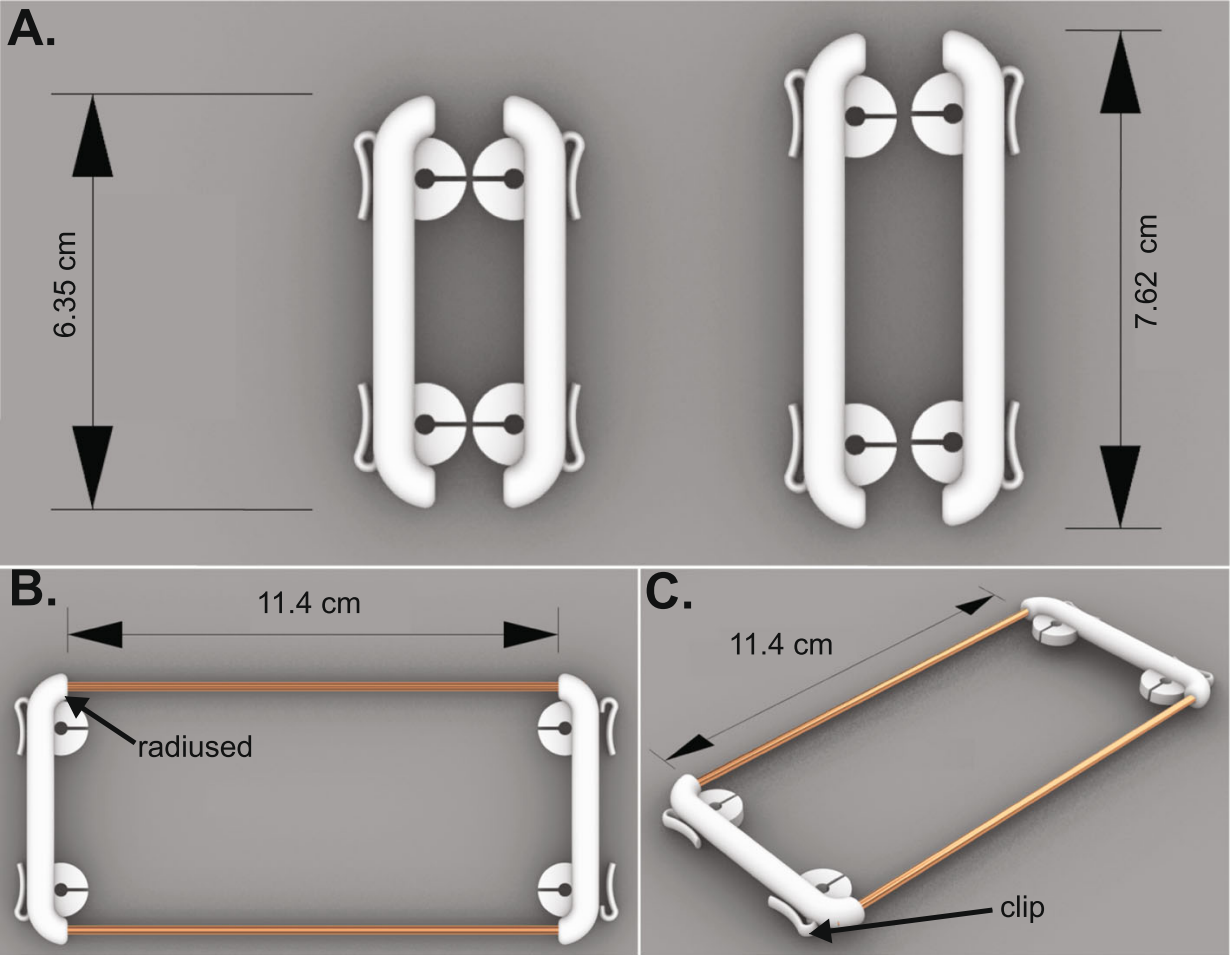
Mask frame components. **a** PLA lateral frames in two sizes: the small size is 6.35 cm long and regular size is 7.62 cm long. **b **and** c** Assembled mask frames consisting of both mask frame and malleable wire (copper, steel, or aluminum). Note that this mask frame involves attaching 3D printed components to wire using cyanoacrylate “super glue”. A mechanical attachment method is described in Fig. 2

3D printed mask frames were designed in Rhinoceros^(R)^ Rhino 6 (Fig. 1) in two sizes. A 3D model (0.3 dm) of the lateral frames was exported in Rhino 6 to a Standard Tessellation Language (.STL) file. The .STL was uploaded to 3dPrinterOS, a cloud-based 3D printing service. 3dPrinterOS converted the .STL to a G-code file, which contains machine commands that control the 3D printers’ movement and deposition of material, which was then sent to a 3D printer. Print settings were chosen by using the default values for the 3dPrinterOS customized for the Dremel 3d45 3D printer, including a print nozzle temperature of 230 °C and a print bed temperature of 60 °C. Other printer settings included a standard layer height of .3mm, a 1.2mm sidewall shell thickness, 10 % infill in a ‘grid’ pattern, and a top and bottom layer shell thickness of 2mm.

**Fig. 2:**
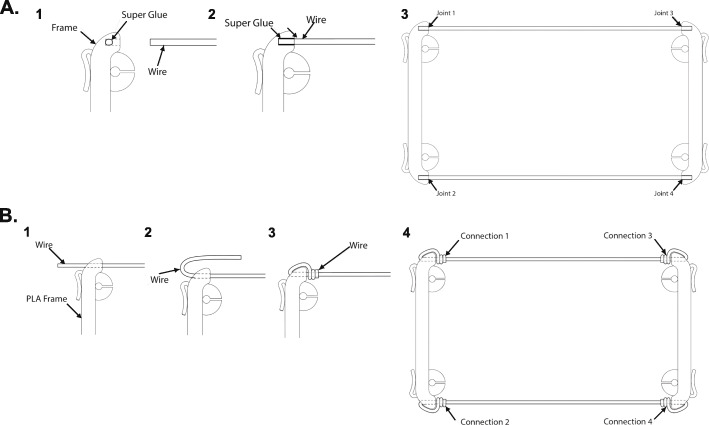
Methods for mask frame assembly. **a** Method 1 for mask frame assembly utilizing glue adhesive. (1) One drop of cyanoacrylate super glue is placed into the end slot for the wire within the PLA lateral frame. (2) The end of a wire is inserted into the slot. (3) All four wire ends are inserted into the PLA slots as shown to complete the frame. **b** Method 2 for mask frame assembly using wire alone (no adhesive). (1) Wire is pushed through the opening in the PLA lateral frame. (2) The wire is looped back and (3) twisted around itself using pliers. (4) This process is repeated for each of the four total connections

### 3D printer model and hardware

Dremel-branded 1.75 mm diameter PLA was used in a compact consumer-grade Dremel 3d45 3D printer for all frames. The printer had a .4mm nozzle extrusion width and a build volume of 254 × 152 × 170 mm. Print time for one regular sized mask frame was approximately 30 min.

### Mask frame assembly

Two methods of mask frame assembly were developed, each of which involved a slight modification to the 3D printed lateral frame. Method 1 (Fig. 2a) uses an adhesive, cyanoacrylate (“super glue”), to join the mask frame components; the prototype design is shown in Fig. 1. The Method 1 assembly sequence is as follows: gather components (2 flexible wires cut to 127 mm, 2 PLA lateral frames, and 1 bottle of super glue). (1) Place one drop of super glue in the lateral frame joint. (2) Insert wire into joint. Follow instructions accompanying super glue for holding wire in place to properly allow the glue to set and cure. (3) Repeat for each joint.

Method 2 (Fig. 2b) involves a mechanical connection in which formable wire is twisted to join mask frame components. Method 2 assembly sequence is as follows: gather components (2 flexible wires cut to 195 mm and two PLA lateral frames that have a hole through the joint). (1) Push wire through the joint in the lateral frame. (2) Loop wire back. (3) Twist wire around itself. Use of pliers is recommended to assist in bending and twisting of wire to ensure a secure twist. (4) Repeat for each connection. Widely available solid wire, often used for electrical cable, works well in this application.

### Band and clip attachment

**Fig. 3:**
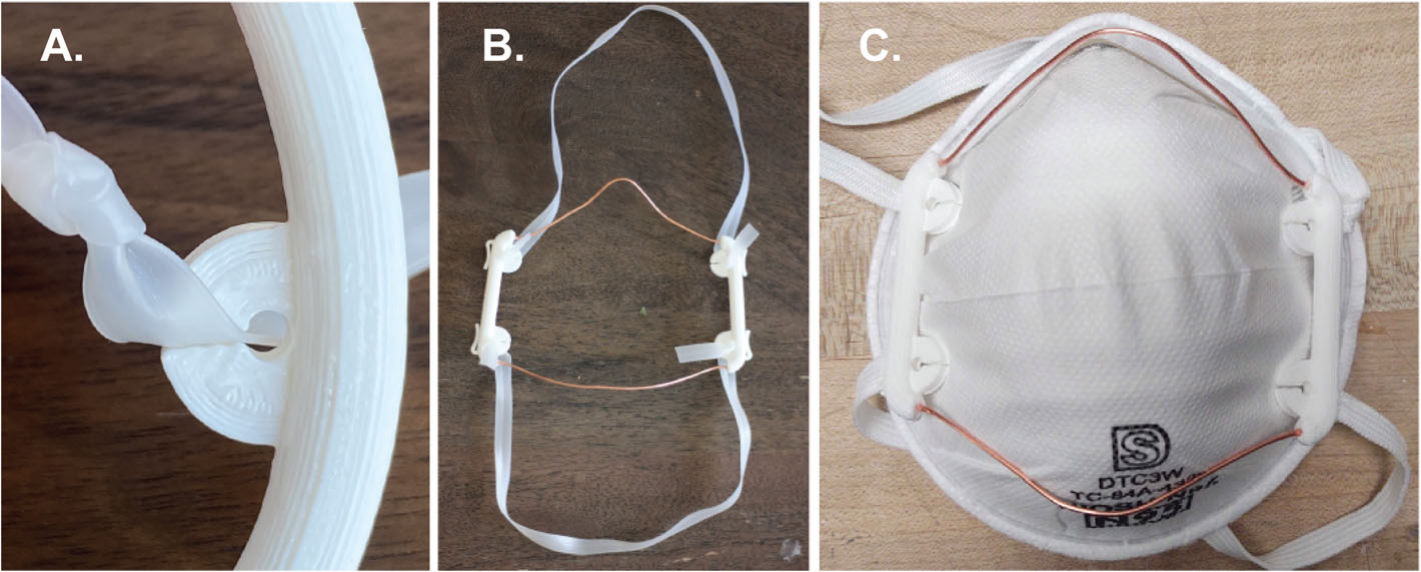
Attachment of the head band to a mask frame. **a** A knot is tied at the end of each band and the band is then slid into and locked in place using the PLA slot. **b** Mask frame with bands. **c** Clip attachment of the frame to the bands on the 3M N95 Model 1860 respirator

Based on prior work creating 3D printed mask frames by colleagues at the University of Connecticut [17], the band material used for this study was Monprene^(R)^ PR-23040 in the following size: 0.25 in x 0.015 in (Teknor Apex; Pawtucket, RI). Two strips of elastic 305 mm and 330 mm in length were cut for the 1860 and KN95 masks, two strips 356 mm and 381 mm in length were cut for the 8210, and two strips 254 mm and 279 mm in length were cut for the duckbill. A knot was tied at each end of each band, approximately 25 mm from the end of the band. The knot was locked into each slot in the PLA frame as shown in Fig. 3a and b. The clips along the PLA frame were attached to existing bands of the respirator, if still present, to secure the frame to the mask (Fig. 3c).

### Donning, doffing and sterilization

**Fig. 4:**
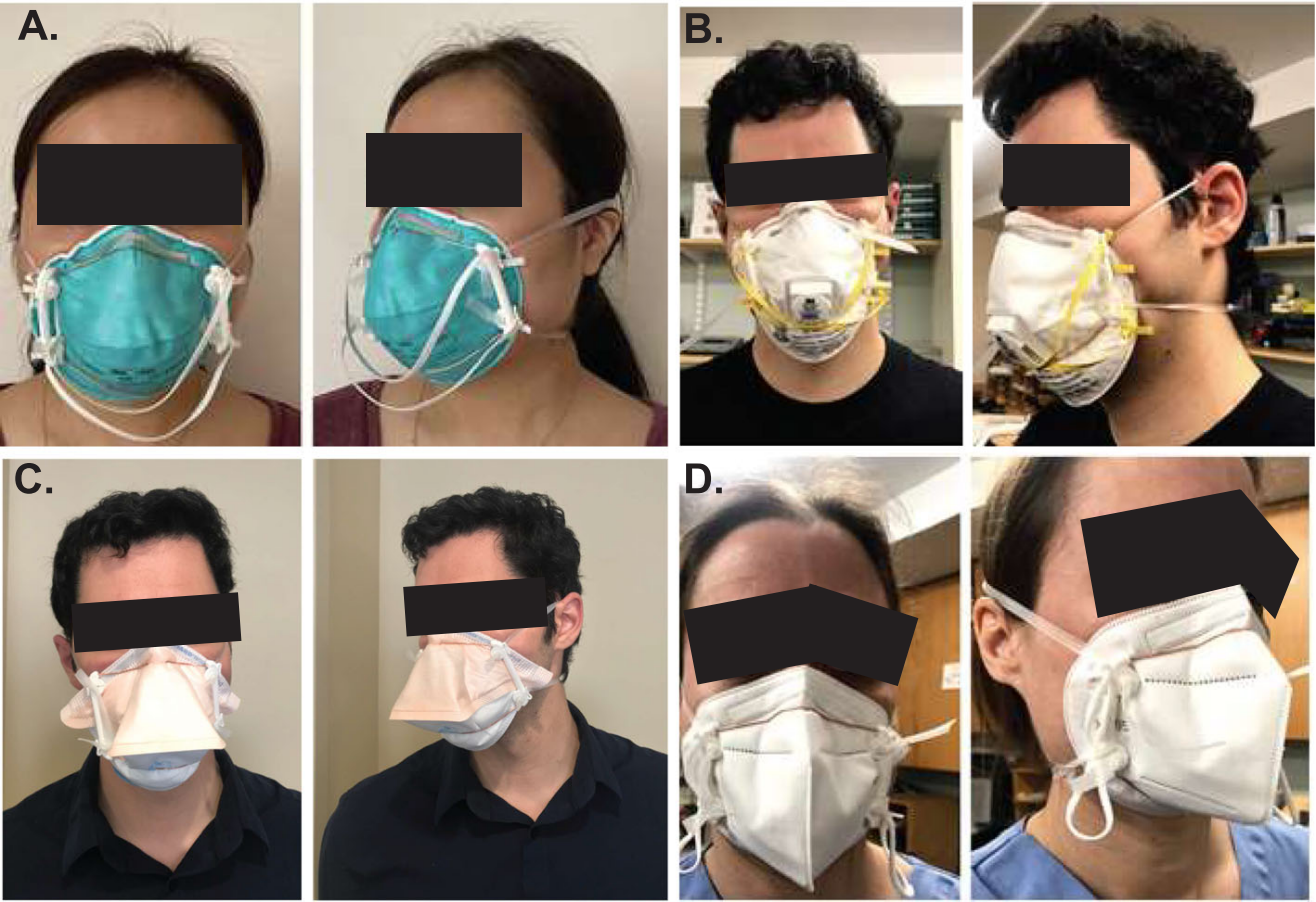
Properly donned mask frames and respirators on three different volunteers. **a** A 3M 1860 N95 domed healthcare respirator, **b** a 3M 8210 N95 domed industrial respirator (note a valve-less version of the model is used in healthcare settings but was not always available for testing due to widespread respirator shortages), **c** a Kimberly Clark duckbill, and **d** a KN95 flat-fold respirator. The bands should be sufficiently tight and the nosepiece manipulated to achieve a good seal

Once the mask frame is attached to the respirator using the clips, the respirator is donned just like a respirator without a frame. Holding the respirator and mask frame in the palms of two hands, the respirator and then the frame is brought to the face to cover the nose and mouth; the mask frame should not pass outside the borders of the mask. The bottom strap attached to the mask frame is brought up and over the top of the head and placed at the nape of the neck below the ears. The upper strap is pulled up behind the head and placed at the crown of the head. Then, the nosepiece of the mask frame is manipulated in the shape of the user’s nose until a secure seal and good fit are achieved (Fig. 4). A seal check is performed by placing both hands over the mask and inhaling, then exhaling. The mask should first collapse slightly; during exhalation, if air leakage is observed, the fit is inadequate and the nosepiece and wires should be adjusted and straps pulled tighter until a proper seal is obtained. For masks passing seal check, fit was then tested using qualitative and quantitative fit testing apparatus and methods [19]. Mask frames can be sterilized using 70 % isopropyl alcohol wipes.

### Selection of FFRs for testing

Four different types of N95-style FFRs were selected for testing: a 3M Model 1860 N95, a 3M Model 8210 N95, a Kimberly Clark 46827/46767 (hereafter referred to as “duckbill”), and a Cooper KN95 (imprinted with number XK02-001-00010; Cooper USA; Las Vegas, NV). The 3M model 1860, available in both small and regular sizes, is a standard dome or cup-shaped respirator commonly used in healthcare settings [20] and is fabricated from media (fabric) that provides enhanced fluid and splash resistance (per ASTM Test Method F1862 [21]). The Kimberly Clark regular (46767) and small (46827) models, like the 3M 1860 model, were used in healthcare settings prior to the pandemic and are fabricated according to ASTM standards but are duckbill instead of dome-shaped. We also tested an industrial 3M model 8210 mask, only available in a single standard size, that would not normally be used in a healthcare setting but whose temporary use is permitted in the US under an FDA Emergency Use Authorization (EUA) issued on April 3, 2020 [22]. The Cooper flat-fold KN95 respirator, available in one standard size, was selected as prototypical of a non-US manufactured FFR whose use in healthcare is also allowed by an FDA EUA. With KN95 masks it has been observed that even when filtration efficiency meets specifications, fit can be problematic [23, 24].

### Test subject demographics

A total of 45 volunteers were involved in this study and consisted of attending physicians, resident physicians, clinical fellows, medical students, nurses, medical assistants, clinic staff, and research scientists, with predominantly female participants (making up 60–88 % of each test group; Table 1; raw data presented in Additional Material 1). The proportion of female participants is representative of the healthcare workforce, which is predominantly female in the US and worldwide [25]. Of note, prior literature shows that women fail fit testing approximately 10 % more frequently than men [26], suggesting a greater potential need for methods to improve fit with female FFR users. Individuals had a BMI ranging from 18.5 to 56.6 (averaging 25.4 for all groups).

**Table 1:** Demographics and characteristics of participants undergoing baseline fit testing. SD = standard deviation. KC denotes Kimberly-Clark Inc

	**No. (%) or Mean +/- SD (range)**			
**Characteristic**	**1860 N95 respirators (n=34)**	**8210 N95 respirators (n=10)**	**KC duckbill respirators (n=16)**	**KN95 respirators (n=25)**
**Sex**				
Female	28 (82.4%)	6 (60.0%)	14 (87.5%)	22 (88.0%)
Male	6 (17.6%)	4 (40.0%)	2 (12.5%)	3 (12.0%)
**Age**	36.4 +/- 10.3	32.9 +/- 9.1	33.1 +/- 9.3	36.5 +/- 12.5
**Ethnicity**				
Asian/Pacific Islander	11 (32.4%)	3 (30.0%)	2 (12.5%)	5 (20.0%)
White/Caucasian	11 (32.4%)	7 (70.0%)	3 (18.8%)	8 (32.0%)
Black/African American	8 (23.5%)	0 (0.0%)	8 (50.0 %)	9 (36.0%)
Hispanic/Latino	3 (8.8%)	0 (0.0%)	2 (12.5%)	3 (12.0%)
Native American	1 (2.9%)	0 (0.0%)	1 (6.3%)	0 (0.0%)
**Body mass index (BMI)**	25.7 +/- 8.0	23.3 +/-2.7	30.1 +/- 10.0	26.7 +/- 8.5
**Healthcare role**				
Attending physician	15 (44.1%)	2 (20.0%)	1 (6.3%)	6 (24.0%)
Resident physician or fellow	1 (2.9%)	0 (0.0%)	0 (0.0%)	2 (8.0%)
Medical student	3 (8.8%)	3 (30.0%)	0 (0.0%)	2 (8.0%)
Researcher	5 (14.7%)	5 (50.0%)	3 (18.8%)	1 (4.0%)
Nurse	3 (8.8%)	0 (0.0%)	3 (18.8%)	5 (20.0%)
Medical Assistant	5 (14.7%)	0 (0.0%)	5 (31.3%)	5 (20.0%)
Clinic Staff	2 (5.9%)	0 (0.0%)	4 (25.0%)	4 (16.0%)
**Mask size (previously fitted)**				
Small	11 (32.4%)	6 (60.0%)	3 (18.8%)	6 (24.0%)
Regular	23 (67.6%)	4 (40.0%)	13 (81.3%)	19 (76.0%)
**Baseline Testing Pass Rate**	30/34 (88.2%)	9/10 (90.0%)	15/16 (93.8%)	12/25 (48.0%)

### Qualitative and quantitative fit testing

**Fig. 5:**
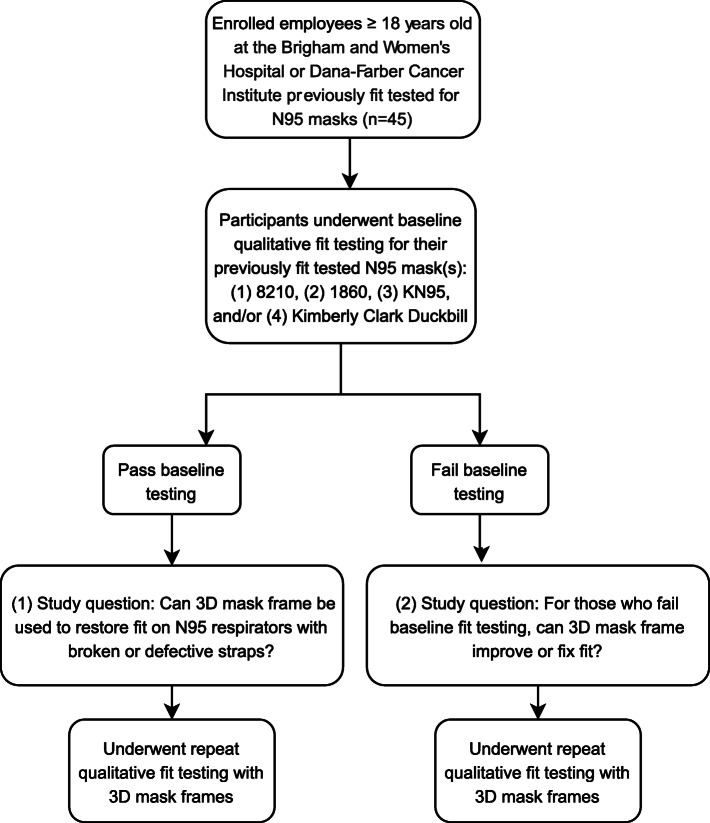
Flow chart of study qualitative fit testing methods

 This study was approved by the Partners Healthcare Institutional Review Board (protocol #2020P001209). All subjects underwent qualitative fit testing at the Brigham and Women’s Hospital or Dana-Farber Cancer Institute during May 2020-March 2021. Qualitative fit testing using a 3M FT-14 hood and 3M FT-32 bitter testing solution was performed over two different testing sessions, both consisting of tests without the mask frame (baseline) and with the 3D printed mask frame (Fig. 5). Qualitative fit failure occurred if the participant could taste the solution (bitter taste). Four different models of respirators were tested: 1860, 8210, duckbill, and KN95. Data was analyzed using Prism version 8 (GraphPad, San Diego, CA). Quantitative fit testing was also performed on the respirators with the mask frame in place. Quantitative fit testing employed a non-hazardous aerosol in a test chamber (PortaCount, TSI Inc., Shoreview, MN) to measure the volumetric leak rate and to calculate the respirator fit factor (FF). The FF is the ratio of the average chamber aerosol concentration compared to the concentration inside the respirator, with a passing value consisting of an FF above 100. Three different models of respirators were tested: 3M Model 1860 N95, 3M Model 8210 N95, and Cooper KN95.

## Results

### Frame design and fabrication

The mask frame we developed consisted of two malleable wire components (made of copper, steel, or aluminum) that link together two rigid lateral PLA frames fabricated by 3D printing (Fig. 1). A total of six weeks was required to design, prototype, and fabricate mask frames for testing (additional information on the iterative design process can be found in Methods). The resulting design was flexible enough to conform to a diversity of face types and sizes but rigid enough to seal masks to users’ faces. The malleable wires enable the mask frame to be molded to each user’s face, creating a personalized fit unique to individual facial contours and respirator models (Fig. 4). Frames were fabricated on a standard consumer grade 3D printer in less than 30 min at a cost of approximately 0.50 USD. Two sizes of lateral frames were printed in PLA and made available to participants: a “small” size (6.35 cm long) and a “regular” size (7.62 cm long) (Fig. 1a). Additional features included radiused edges on the lateral frame to prevent the N95 FFR from being excessively deformed **(**Fig. 1b**)**; deformation was observed with prototypes in which the edges were square or “V” shaped. We tested two different methods of attaching the malleable wire, one that used adhesive (Fig. 2a**)** and a second that involved mechanical twisting (Fig. 2b). The method of wire attachment did not detectably affect comfort or function but slight design modifications were necessary to accommodate 3D printed frames to the two attachment methods. As a source of replacement elastic straps, we used a non-latex material (Monprene^(R)^ PR-23040) which is widely available, FDA-approved, and used for phlebotomy tourniquets. All design files and testing results are included in this manuscript and available for reuse without restriction; design information is also available via the NIH 3D Print Exchange (https://3dprint.nih.gov/discover/3dpx-014725).

**Fig. 6:**
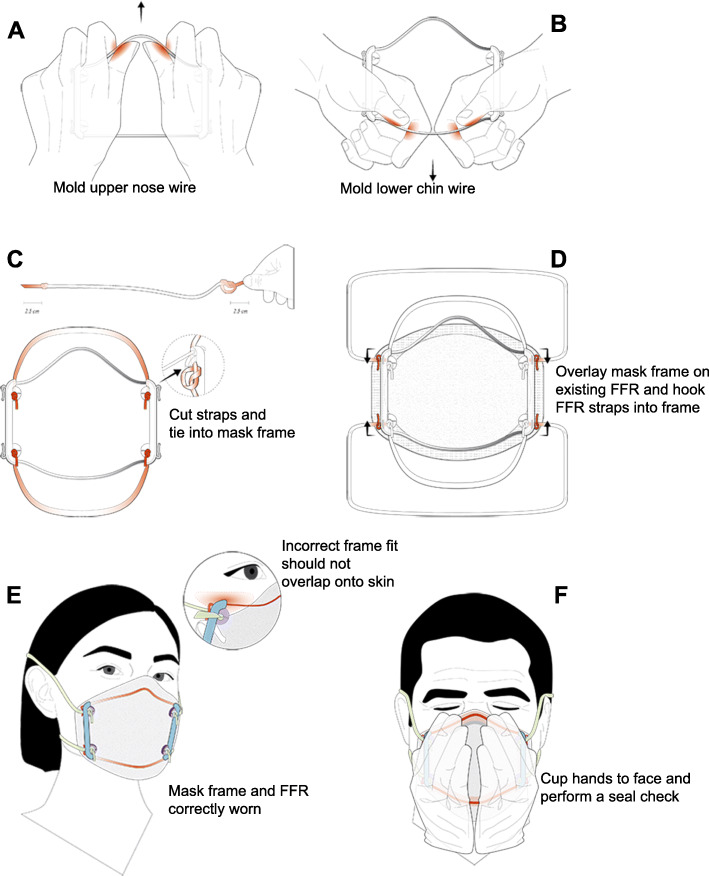
Mask frame assembly instructions

In use, elastic straps lock into slots on each side of the 3D printed piece and are cut to a standardized length that fits around a users’ head, just like factory-supplied straps (Fig. 3a, b). Additional clips along the frame make it possible to secure any remaining respirator bands to the frame. By adjusting the position of the strap in the frame slots it is possible to adjust the pressure holding the mask in place and achieve a good but comfortable seal (Figs. 1c, 3c). A frame with an attached mask is donned just like a mask without a frame: the respirator is brought to the face to cover the nose and mouth, the lower strap is brought up and over the top of the head and the upper strap is pulled up behind the head. The nosepiece of the mask frame is then press-fit, or otherwise bent, to conform to shape of a user’s nose (Fig. 6; additional fitting, assembly, and donning instructions are described in detail in Additional Material 2; example fits are shown in Fig. 4). Individual users were allowed to choose a frame size based on whether their faces were “small” or “regular.”

### Fit testing

Among our group of 45 volunteers, 34 were fit-tested with 3M model 1860 masks, 10 with 3M 8210, 25 with Cooper KN95 masks, and 16 with the Kimberly Clark duckbills. It was not possible to test all masks on all individuals due to severely limited supply of healthcare FFRs resulting from the COVID-19 pandemic (each mask could only be tested on a single individual for safety reasons). As a result, volunteers were asked to select a mask and mask frame size based on previous experience in a clinical setting (if available) (Table 1). Qualitative fit testing was performed using a 3M FT-14 standardized hood and 3M FT-32 bitter testing solution; if a user could taste the aerosolized fluid, the fit test was judged to have failed. Fit was tested without a mask frame (the baseline condition) and with the 3D printed mask frame in place of the mask straps (the test condition; outlined in Fig. 5). For 3M 8210 masks and Kimberly Clark duckbills, 9/10 (90.0 %) and 15/16 (93.8 %) of participants passed baseline testing without a 3D printed mask frame, respectively. For the 3M 1860 and KN95 masks, baseline passing rates were lower at 30/34 (88.2 %) and 12/25 (48.0 %), respectively. The passing rates for the 1860, duckbill, and 8210 models are consistent with previous literature demonstrating 82–95 % fit rates across N95 respirator models [26, 27]. Fit for KN95 masks was poorer, as previously reported [16].

We then asked whether individuals for whom a mask passed qualitative fit testing under baseline conditions would also pass fit testing when elastic straps were removed (or allowed to hang down) and the masks held in place only using a frame and replacement straps. We note that masks lacking straps are normally unusable and thus, 0 % would be able to pass testing. For model 8210 (Table 2; Fig. 4b), 100 % of participants who passed qualitative fit testing at baseline preserved fit using a mask frame in the broken or defective strap test (9/9). For individuals who passed fit testing with a model 1860 mask (Table 2; Fig. 4a), a Kimberly Clark duckbill (Table 2; Fig. 4c), or a KN95 mask (Table 2; Fig. 4d), the passing rates with the 3D mask frame in the broken or defective strap test were 24/30 (80.0 %), 12/15 (80.0 %), and 11/12 (91.7 %) respectively (raw data presented in Additional Material 1).

**Table 2:** Qualitative fit testing results using mask frames

**Type of test:**	**Performance of frames with respirators with broken or defective straps**	**Performance of frames for individuals who failed baseline testing**
**1860 model (total n = 34)**		
Number Passed (%)	24/30 (80.0 %)	0/4 (0 %)
**8210 model (total n = 10)**		
Number Passed (%)	9/9 (100 %)	0/1 (0 %)
**Kimberly Clark duckbill model (total n = 16)**		
Number Passed (%)	12/15 (80.0 %)	0/1 (0 %)
**KN95 model (total n = 25)**		
Number Passed (%)	11/12 (91.7 %)	6/13 (46.2 %)

We also asked whether mask frames would improve fit for participants who failed baseline qualitative testing. For N95 masks, the relatively small number of individuals who failed made it difficult to obtain statistically robust results. All participants who failed baseline testing for the three models of N95 masks also failed to pass qualitative fit testing using a frame including for 1860 masks (0/4 participants passed), 8210 masks (the single participant did not pass), and Kimberly Clark duckbills (the single participant did not pass). The most promising data were obtained with KN95 masks. Among the 13 individuals for whom a KN95 did not pass qualitative fit testing, six (46.2 %) achieved an acceptable fit with a frame (Table 2). We also conducted limited quantitative fit testing on 1860, 8210, and KN95 masks (Table 3). The quantitative fit test results with frames were similar to the qualitative fit test passing rates: 1/1 passing for each of the 1860 and 8210 models and 1/2 (50.0 %) passing with a KN95 model. These results suggest that it may be possible to use frames to overcome previously noted problems in achieving adequate fit with KN95 masks.

## Discussion

In this paper we describe an iterative design process, involving multiple rounds of prototyping, clinical feedback, and design modification that resulted in a simple mask frame consisting of two identical 3D printed components (made in two sizes to accommodate different faces) and two pieces of malleable wire that together hold an N95 or KN95 mask to users’ faces in the absence of factory-supplied straps or following repeated donning and doffing cycles that interfere with fit. Such mask frames are reusable and can be sterilized using 70 % isopropyl alcohol wipes. Across a diverse group of volunteers, we found that mask frames were effective in replacing the original straps on all three N95 masks and one KN95 model tested. The ability to replace masks degraded with age or by multiple donning/doffing cycles has the potential to immediately impact PPE supplies for healthcare workers at low cost and complexity. Other approaches have been proposed for specifically for replacing defective straps, including sewing new elastic bands directly to the respirator material [28]. We are not aware of any testing performed on this approach, but when the pandemic recedes, medical grade FFRs once again become widely available, and demand on fit-testing apparatus falls, a systematic comparison of different ways to reuse damaged or degraded FFRs will become feasible. Such a study will be valuable for future emergencies. Until then, each group should perform its own evaluation by fit testing users who will use mask frames or replacement straps in a clinical setting.

Results were promising but not fully conclusive with respect to our additional goal of improving fit for participants who failed baseline qualitative fit-testing: frames were effective for some individuals and mask models and ineffective in other cases. The most promising results were obtained with KN95 flat-fold masks, for which achieving a good fit is known to be challenging [16]. Among individuals for whom a KN95 did not pass fit testing, ~ 50 % achieved an acceptable fit as judged by both quantitative and qualitative fit testing criteria (e.g. using a PortaCount quantitative fit testing apparatus, TSI Inc., Shoreview, MN). When this is combined with individuals for whom KN95 masks fit without any modification, around three-quarters of individuals tested could use a KN95 mask in a clinical setting. Because KN95 masks are widely available and in use for respiratory protection in medical settings [29], this aspect of our work represents a contribution of immediate practical value and goes beyond simply replacing defective straps. Existing studies have described modifications that improve the fit of cloth or procedural masks [30] but to our knowledge we are the first to develop and test a product that improves KN95 fit for a substantial fraction of individuals. We anticipate that with additional modifications, some as simple as developing frames specifically for narrow faces, or creating a phone-based image processing tool for optimizing wire length for different faces, it will be possible to further extend our findings to solve long-standing problems in FFR fit.

Multiple recent projects have attempted to develop reusable respirators to replace disposable N95s, especially within the global 3D printing community. For instance, the Copper3D NanoHack mask is printed with a PLA filament as a flat piece, and is manually assembled into a 3D configuration using hot air (e.g. hairdryer) or hot water [31]; two reusable filter cartridges are then inserted into an intake port. The HEPA Mask [32] and the Lowell Makes Mask [33] both involve similar 3D printed components in PLA but with different variations in the filter holders. The Injection Molded Autoclavable, Scalable, Conformable (iMASC) is constructed with injection-molded liquid silicone rubber (Table 4). There is little data evaluating the fit and seal for most of FFR 3D printed alternatives. Of the alternative mask frame designs, the only study with available fit testing data assessed the improvement of fit for procedural masks (Table 4). None of the surveyed alternative designs involve improving the fit and seal of commercially available N95-type masks. The issue of fit has been tackled in creative ways, including by experimenting with flexible materials or surface scanning an intended user’s face and creating a custom-fit device (such as the World Advanced Saving Project (WASP) [34] and Bellus3D [35]). A limitation in all of these approaches is that fabrication is complex, limiting throughput [36, 37], and in some cases supplies of the necessary filters have been largely depleted, making it challenging to produce and distribute these products during a medical supply shortage [5]. A more fundamental problem is that, in the absence of extensive testing across a wide range of conditions, it remains unknown how well alternative filter media will compare to the thoroughly validated electret fabric used in N95 and KN95 masks. Conventional N95 masks are therefore likely to remain the primary form of respiratory PPE during the current pandemic. It is therefore reasonable to focus on improving N95 FFRs using simple supplementary devices that do not affect filtration properties.

**Table 3:** Quantitative fit testing results using mask frames

**Performance of frames with respirators with broken or defective straps**		
**Mask type**	**Fit factor with control mask **	**Fit factor with mask frame on respirator with broken or defective straps**
1860 model	185	200+
1860 model	13	9
8210 model	17	19
**Performance of frames for individuals who failed baseline testing**		
**Mask type**	**Fit factor with control mask **	**Fit factor with mask frame on respirator** **that failed baseline testing**
1860 model	56	200+
8210 model	0	200+
KN95 model	0	101
KN95 model	0	6

Testing was performed on previously used and sterilized respirators due to supply shortages in the setting of the COVID-19 pandemic. Control testing was performed for each respirator without the mask frame but with the use of functional straps. A fit factor of 100 is considered a passing value

**Table 4:** Alternative mask and mask frame design solutions developed to address shortages in N95-tyle masks caused by the COVID pandemic

**Design Name**	**Description**	**Fit Testing Data Available**
Copper3D NanoHack	De-novo mask design. 3D printed mask with filter media made of non-woven polypropylene impregnated with 5% copper oxide particles.	No [31]
HEPA Mask	De-novo mask design. 3D printed mask to house commercial HEPA filter insert.	No [32]
Injection Molded Autoclavable, Scalable, Conformable (iMASC) system	De-novo mask design. Liquid silicone rubber mask using filters cut from 3M 1860 N95 FFRs.	Yes [38]
Lowell Makes Mask	De-novo mask design. 3D printed mask with customizable filter insert.	No [33]
WASP My Face MaskBellus 3D	De-novo mask design. 3D printed mask with customizable filter insert.	No [34]
Bellus 3D	Mask frame design. Rigid 3D printed mask frame that fits over existing mask; uses picture of user’s face to personalize printed design.	No [35]
Double Eight Masks Brace	Mask frame design. Rubber band brace used over existing surgical mask.	Yes [39]
PanFab Mask Frame	Mask frame in two sizes for use with multiple models of N95 and KN95 masks to replace defective straps or damaged nose pieces.	Yes (this work)

How great is the need for extending usable mask life using a device such as the frame described here? Historical guidance by NIOSH specifies that the useful lifetime of NIOSH-approved FFRs is limited by filter load and that any filter or mask should be replaced if it becomes soiled, damaged, or causes noticeably increased breathing resistance [40]. In environments that generate high cumulative filter loading, the recommended maximum lifespan for N95 respirators is eight hours, and it is standard practice in healthcare to dispose of N95 masks after each patient encounter. However, during the first SARS pandemic, the CDC stated that “health care facilities may consider reuse as long as the device has not been obviously soiled or damaged (e.g. creased or torn)” and “if a sufficient supply of respirators is not available [41].” Multiple mask decontamination methods were tested and implemented during the COVID-19 pandemic and have further expanded on this concept, including UV germicidal irradiation, vaporous and ionized hydrogen peroxide (VHP/iHP) and moist heat [42–49], all of which promise to enable N95 reuse (for instance, the FDA EUA for the Battelle decontamination system permits up to 20 vaporous hydrogen peroxide decontamination cycles per respirator [50]). However, mask reuse is generally limited by breakage or degradation of the malleable nose clips and other components that are bent to form a seal around a user’s nose and the elastic bands that hold a mask in place. It has also been reported that N95 masks stored in preparation for a pandemic have a high rate of band failure [14].

Quantitative fit testing has shown that multiple donnings and doffings degrade mask fit independent of band failure. Bergman et al. [12] found that, after five consecutive donnings, fit factors consistently dropped to failing values. Vuma et al. [51] showed that, when 25 tests subjects performed consecutive N95 donning and doffing operations, fit factor differed significantly between the first and the sixth re-donnings. After the sixth donning, only ~ 68 % of study subjects achieved a passing fit. Degsys et al. [52] found that an increase in fit failures was associated with an increasing number of shifts, each of which was associated with a donning and doffing cycle (median 4 shifts) and increasing hours of use (median 14 h). Additionally, mask fit is adversely affected by repeated cycles of decontamination across a variety of methods (including heat and ethanol) [53]. If undetected, poor fit causes air to flow around the mask [14] potentially increasing disease transmission.

Problems with fit are not restricted to masks that are being reused: it has previously been reported that even with new masks, about 17 % of users will fail fit testing with any specific model of N95 or N95-equivalent mask [27]. The fit failure rate for KN95s has not been extensively quantified in the literature, but available studies suggest that fit failure is an issue with a majority of KN95 models [16]. Improving fit by using a frame would therefore be helpful even in non-pandemic situations. The problem with failure to fit any mask is made worse in a pandemic by shortages in alternative forms of respiratory protection (e.g., powered air-purifying respirators [54, 55]). Thus, both pandemic and non-pandemic respiratory protection presents a substantial need for devices – such as the mask frames described here - to extend the useful lifetime of FFRs, such as N95 or KN95 masks, or improve fit of new masks. It must be noted, however, that clinical data supporting extended use and reuse of N95 masks, with or without decontamination, remains limited. Concerns about extended use and reuse involve not only fit and the adequacy of the seal to a user’s face [52], but also potential infection risks acquired during donning/doffing (since the outer surface of respirators can be contaminated with infectious agents that can be transferred to a user [56]) and reductions in filtration efficiency over time. The mask frames described here address only the first of these issues.

### Limitations of this study

This study has several limitations; most notably, that sample sizes and the number of mask models tested are small. These limitations reflect a continuing shortage in FFRs of all types and our inability to divert more than a small number of masks from hospital or staff use to a research project. Results would be improved by finding a much larger number of participants who failed baseline fit testing and from whom multiple models of masks could be evaluated with and without frames. For all of the masks used in this study, straps were artificially broken or allowed to hang free; to better represent the real-world use case it will be necessary to perform fit testing with and without a mask frame after actual use in a clinical setting. Our study was also limited by an inability to perform direct comparison between mask frames and solutions proposed by others (Table 4). Finally, our data suggest that the precise shape of a mask and the properties of the material may determine whether a frame can successfully substitute for original straps or improve fit for a specific individual. Additional research will be required to identify these variables and address them. Ideally, all of these issues will become increasingly possible to address as supply disruptions recede and sufficient FFRs can be dedicated to research studies.

## Conclusions

As a result of a collaboration with Partners in Health, PanFab mask frames in two sizes are currently in use in the field in Mexico, Malawi, and Haiti with appropriate fit testing in place; we hope to be able to report on field testing in the future. Our results from studies in the US with volunteers clearly show that frames can substitute for broken or defective bands without adversely affecting fit and also improve fit for a substantial number of individuals who cannot use KN95 masks. Because degradation in fit is a limiting factor in extended mask use and reuse of masks after decontamination, mask frames have the potential to help offset the urgent need for respirators encountered at the outset of the COVID-19 and potential future medical emergencies.

## Supplementary Information

**Additional Material 1:** Individual participant data used to construct Tables 1 and 2.

**Additional Material 2:** Mask frame assembly, donning, and seal check instructions with accompanying diagrams.

## Data Availability

All data generated or analyzed during this study are included in this published article and its supplementary information files.

## Abbreviations

3D: 3 Dimensional
FFRs: Filtering Facepiece Respirators
IRB: Institutional Review Board
PLA: Polylactic Acid
PPE: Personal Protective Equipment
