# Supplementary material for: 3D Printed frames to enable reuse and improve the fit of N95 and KN95 respirators
Source: BMC Biomed Eng. 2021 Jun 7;3:10. doi: 10.1186/s42490-021-00055-7 (PMC8182357; doi:10.1186/s42490-021-00055-7)
Supplement: Supplementary file 1 — Additional Material 1: Individual participant data used to construct Tables 1 and 2. [file 42490_2021_55_MOESM1_ESM.docx]

**Additional Material 1: Individual participant data used to construct Tables 1 and 2**

1. Demographics and characteristics of participants undergoing baseline fit testing (n=45)

| **Participant #** | **Baseline Mask Size** | | **Sex** | | **Ethnicity** | **Clinical Role** | |
| --- | --- | --- | --- | --- | --- | --- | --- |
| 1 | Small | | Female | | Asian/Pacific Islander | Attending | |
| 2 | Small | | Female | | White/Caucasian | Attending | |
| 3 | Regular | | Male | | Asian/Pacific Islander | Attending | |
| 4 | Regular | | Female | | White/Caucasian | Attending | |
| 5 | Regular | | Female | | White/Caucasian | Attending | |
| 6 | Regular | | Female | | White/Caucasian | Attending | |
| 7 | Small | | Female | | Asian/Pacific Islander | Attending | |
| 8 | Regular | | Male | | Asian/Pacific Islander | Attending | |
| 9 | Regular | | Female | | White/Caucasian | Attending | |
| 10 | Small | | Female | | White/Caucasian | Attending | |
| 11 | Regular | | Female | | Asian/Pacific Islander | Attending | |
| 12 | Regular | | Female | | Black/African American | Attending | |
| 13 | Regular | | Female | | Asian/Pacific Islander | Attending | |
| 14 | Small | Female | | White/Caucasian | | Attending |  |
| 15 | Regular | Female | | White/Caucasian | | Attending |  |
| 16 | Regular | Female | | Black/African American | | Nurse |  |
| 17 | Regular | Male | | White/Caucasian | | Researcher |  |
| 18 | Regular | Male | | Hispanic/Latino | | Medical Assistant |  |
| 19 | Regular | Female | | Black/African American | | Medical Assistant |  |
| 20 | Regular | Female | | Black/African American | | Medical Assistant |  |
| 21 | Regular | Male | | White/Caucasian | | Researcher |  |
| 22 | Regular | Female | | Black/African American | | Nurse |  |
| 23 | Regular | Female | | White/Caucasian | | Nurse |  |
| 24 | Small | Female | | Hispanic/Latino | | Nurse |  |
| 25 | Regular | Male | | White/Caucasian | | Resident Physician |  |
| 26 | Small | Female | | Asian/Pacific Islander | | Researcher |  |
| 27 | Regular | Male | | White/Caucasian | | Resident Physician |  |
| 28 | Regular | Female | | Black/African American | | Medical Assistant |  |
| 29 | Regular | Male | | White/Caucasian | | Attending |  |
| 30 | Regular | Male | | White/Caucasian | | Medical Student |  |
| 31 | Regular | Female | | White/Caucasian | | Nurse |  |
| 32 | Regular | Female | | Black/African American | | Clinic Staff |  |
| 33 | Regular | Female | | Black/African American | | Clinic Staff |  |
| 34 | Regular | Male | | White/Caucasian | | Researcher |  |
| 35 | Regular | Female | | White/Caucasian | | Clinic Staff |  |
| 36 | Regular | Female | | Black/African American | | Clinic Staff |  |
| 37 | Regular | Female | | Hispanic/Latino | | Medical Assistant |  |
| 38 | Small | Female | | White/Caucasian | | Researcher |  |
| 39 | Small | Female | | Asian/Pacific Islander | | Medical Student |  |
| 40 | Small | Female | | Asian/Pacific Islander | | Medical Student |  |
| 41 | Small | Female | | Asian/Pacific Islander | | Researcher |  |
| 42 | Regular | Male | | Native American | | Researcher |  |
| 43 | Small | Female | | Asian/Pacific Islander | | Researcher |  |
| 44 | Regular | Male | | Hispanic/Latino | | Clinical Fellow |  |
| 45 | Small | Female | | Asian/Pacific Islander | | Researcher |  |

1. Qualitative fit test results by participant (n=45)

| **Participant #** | **8210: Baseline** | **8210: Frame with broken or defective straps** | **8210: Frame with failed baseline** | **1860: Baseline** | **1860: Frame with broken or defective straps** | **1860: Frame with failed baseline** | **KN95: Baseline** | **KN95: Frame with broken or defective straps** | **KN95: Frame with failed baseline** | **KC duckbill: Baseline** | **KC duckbill: Frame with broken or defective straps** | **KC duckbill: Frame with failed baseline** |
| --- | --- | --- | --- | --- | --- | --- | --- | --- | --- | --- | --- | --- |
| 1 |  |  |  | Pass | Pass |  |  |  |  |  |  |  |
| 2 | Pass | Pass |  | Fail |  | Fail |  |  |  | Pass | Pass |  |
| 3 |  |  |  | Pass | Pass |  |  |  |  |  |  |  |
| 4 |  |  |  | Pass | Pass |  |  |  |  |  |  |  |
| 5 |  |  |  | Pass | Fail |  |  |  |  |  |  |  |
| 6 |  |  |  | Pass | Pass |  |  |  |  |  |  |  |
| 7 |  |  |  | Pass | Pass |  |  |  |  |  |  |  |
| 8 |  |  |  | Pass | Pass |  |  |  |  |  |  |  |
| 9 |  |  |  | Pass | Pass |  |  |  |  |  |  |  |
| 10 | Fail |  | Fail | Fail |  | Fail | Fail |  | Fail |  |  |  |
| 11 |  |  |  | Pass | Fail |  | Fail |  | Pass |  |  |  |
| 12 |  |  |  | Fail |  | Fail | Pass | Pass |  |  |  |  |
| 13 |  |  |  | Pass | Pass |  | Pass | Pass |  |  |  |  |
| 14 |  |  |  | Pass | Pass |  | Fail |  | Pass |  |  |  |
| 15 |  |  |  | Pass | Pass |  | Fail |  | Fail |  |  |  |
| 16 |  |  |  | Pass | Pass |  | Pass | Pass |  | Pass | Fail |  |
| 17 | Pass | Pass |  |  |  |  |  |  |  |  |  |  |
| 18 |  |  |  | Pass | Pass |  | Fail |  | Pass | Pass | Pass |  |
| 19 |  |  |  | Pass | Pass |  | Pass | Pass |  | Pass | Pass |  |
| 20 |  |  |  | Pass | Pass |  | Pass | Fail |  | Pass | Pass |  |
| 21 | Pass | Pass |  |  |  |  |  |  |  |  |  |  |
| 22 |  |  |  | Pass | Fail |  | Fail |  | Fail | Fail |  | Fail |
| 23 |  |  |  |  |  |  | Pass | Pass |  |  |  |  |
| 24 |  |  |  |  |  |  | Fail |  | Fail |  |  |  |
| 25 |  |  |  |  |  |  | Pass | Pass |  |  |  |  |
| 26 | Pass | Pass |  | Pass | Pass |  |  |  |  |  |  |  |
| 27 |  |  |  |  |  |  | Fail |  | Fail |  |  |  |
| 28 |  |  |  | Pass | Pass |  | Fail |  | Fail | Pass | Fail |  |
| 29 |  |  |  |  |  |  |  |  |  |  |  |  |
| 30 | Pass | Pass |  | Pass | Pass |  |  |  |  |  |  |  |
| 31 |  |  |  | Pass | Pass |  | Fail |  | Pass | Pass | Pass |  |
| 32 |  |  |  | Pass | Fail |  | Pass | Pass |  | Pass | Pass |  |
| 33 |  |  |  |  |  |  | Fail |  | Pass | Pass | Fail |  |
| 34 | Pass | Pass |  |  |  |  |  |  |  |  |  |  |
| 35 |  |  |  |  |  |  | Pass | Pass |  | Pass | Pass |  |
| 36 |  |  |  | Pass | Fail |  | Pass | Pass |  | Pass | Pass |  |
| 37 |  |  |  | Pass | Fail |  | Fail |  | Pass | Pass | Pass |  |
| 38 | Pass | Pass |  | Pass | Pass |  |  |  |  |  |  |  |
| 39 | Pass | Pass |  | Pass | Pass |  | Fail |  | Fail |  |  |  |
| 40 | Pass | Pass |  | Pass | Pass |  | Pass | Pass |  |  |  |  |
| 41 |  |  |  | Pass | Pass |  |  |  |  | Pass | Pass |  |
| 42 |  |  |  | Pass | Pass |  |  |  |  | Pass | Pass |  |
| 43 |  |  |  | Fail |  | Fail |  |  |  | Pass | Pass |  |
| 44 |  |  |  | Pass | Pass |  |  |  |  |  |  |  |
| 45 |  |  |  |  |  |  | Pass | Pass |  |  |  |  |
