# Supplementary material for: 3D Printed frames to enable reuse and improve the fit of N95 and KN95 respirators
Source: BMC Biomed Eng. 2021 Jun 7;3:10. doi: 10.1186/s42490-021-00055-7 (PMC8182357; doi:10.1186/s42490-021-00055-7)
Supplement: Supplementary file 2 — Additional Material 2: Mask frame assembly, donning, and seal check instructions with accompanying diagrams. [file 42490_2021_55_MOESM2_ESM.pdf]

3D Printed Mask Frames - Overview and Assembly

OVERVIEW

Diagrammed components

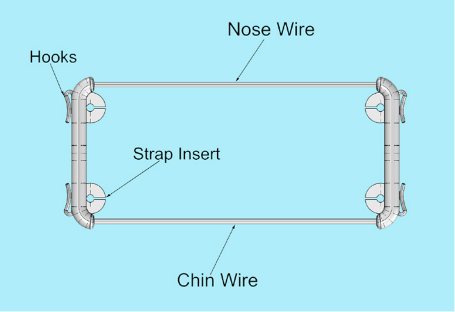

Fully assembled

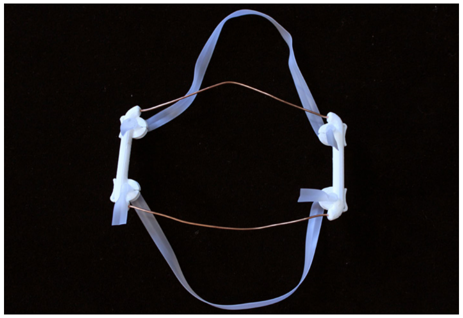

These 3D printed mask frames have been designed to help prolong the lifespan of filtering facepiece respirators, such as N95 masks, during pandemic-level shortage situations. These frames sit on the outside of the mask and conform to the user's face shape to expand the usability of ill-fitting respirators by replacing defective bands and improving fit.

REQUIRED MATERIALS

- mask
- mask frame (small or regular size)
- 2 elastic straps (see below for lengths)

Recommended lengths for Monprene(R) PR-23040 elastic straps

Mask Frame	1860 N95	8210 N95	KN95
Top Strap Length (mm)	330	330	356
Bottom Strap Length (mm)	305	305	381

Note: Straps made out of other materials may require different lengths. In general we recommend smaller strap lengths for more elastic materials, as unsecured mask frames will not improve mask seal.

MASK FRAME ASSEMBLY

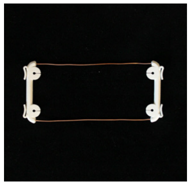

1 **Mask frame sizing instructions:** Choose a mask frame size (small or regular) based on previous respirator fit testing if possible. If you have not been fit tested, estimate frame size based on face size.

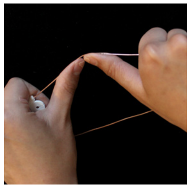

2 **Pre-shape Wires to face shape:** Mold the top wire of the mask frame into the shape of the bridge of your nose ("nose wire") and push down the bottom wire to round it for the chin shape ("chin wire"). Without the mask, place mask frame on your face and shape the nose and chin wires into the shape of your face.

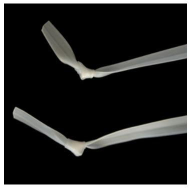

3 **Prepare elastic straps:**

Cut straps: Cut two Monprene(R) PR-23040 elastic straps to desired length by mask type (see Table below) and mark each side 25mm (2.5cm, 1 inch) from either end with a marker (four marks total).

Tie strap knots: Tie a knot at both ends of the two straps at the marker lines.

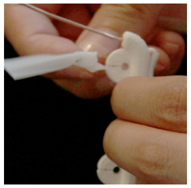

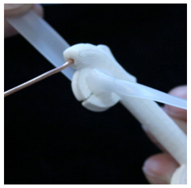

4 **Attach straps:** Take the mask frame and slide the four knotted ends into the four circular strap-holders. There should be one strap on the two top inserts, and the other strap should be on the bottom two inserts. Note that the four knots should all be on the same side of the mask frame (i.e. if the frame is placed on a table, the knots should all be above the frame).

PAN-FAB
GREATER BOSTON PANDEMIC FABRICATION TEAM

3D Printed Mask Frames - Mask Donning & Seal Check

I. DON MASK AND MASK FRAME

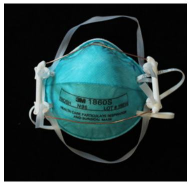

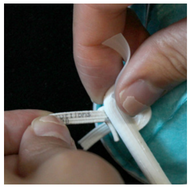

1 **Attach frame to mask**

Overlay frame: Place the mask frame onto the outer surface of the mask, with the strap knots facing away from the mask.

Hook straps: If the original mask still has straps, hook the original mask straps into the four clips on the sides of the mask frame.

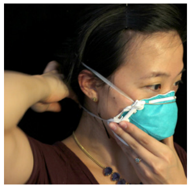

2 **Don mask frame:** While holding the mask and attached mask frame up to your face, pull the bottom mask frame strap behind your head, and then pull the top strap behind your head. This process is the same as typical N95 mask donning procedures.

3 **Evaluate strap tightness:** Check if the mask frame straps are comfortable and tight enough to enable an airtight seal. If not, remove the mask frame and re-knot one side of each strap closer to the middle to shorten the strap.

II. CHECK ALIGNMENT

CORRECT

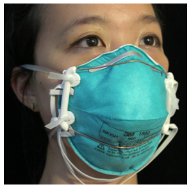

INCORRECT

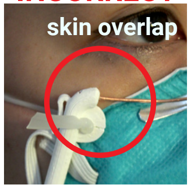

4 **Frame fully overlaps mask:** Frame should sit fully on top of the mask with no contact on skin. If the mask frame touches the skin, adjust the frame until the overlap is complete.

5 **Symmetric overlay:** Ensure mask frame is fully centered on the mask.

III. FORM AND CONFIRM SEAL

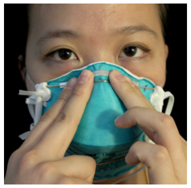

6 **Shape wires to form seal:** To create a tight seal, again mold the nose and chin wires over the mask into the shape of your face.

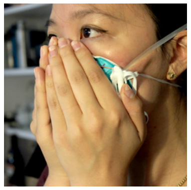

7 **Complete a seal check**

Place both hands over the mask frame, cupping it on both sides. **Do not press down on the mask or mask frame.**

Exhale and check to ensure the respirator bulges slightly; inhale and check to ensure the respirator collapses slightly. Make sure there are no air leaks between the face and respirator.

TROUBLESHOOTING FIT

If the mask frame system does not pass a seal test, check the following to make sure an adequately tight fit has been achieved:

1 **Re-mold** the nose and chin wires to the shape of your face.

2 **Re-check the tightness of the elastic** (may need to be shortened to tighten fit).

3 **Ensure the mask frame** is still centered over the mask and is not in contact with the skin.

4 **If you continue to fail seal check**, try a different size mask frame if available.

More information at https://www.medrxiv.org/content/10.1101/2020.07.20.20151019v1

PAN-FAB
GREATER BOSTON PANDEMIC FABRICATION TEAM
